# Transcriptome studies of congenital heart diseases: identifying current gaps and therapeutic frontiers

**DOI:** 10.3389/fgene.2023.1278747

**Published:** 2023-12-13

**Authors:** Nkechi Martina Odogwu, Clinton Hagen, Timothy J. Nelson

**Affiliations:** ^1^ Program for Hypoplastic Left Heart Syndrome, Mayo Clinic, Rochester, MN, United States; ^2^ Center for Regenerative Medicine, Mayo Clinic, Rochester, MN, United States; ^3^ Division of General Internal Medicine, Mayo Clinic, Rochester, MN, United States; ^4^ Department of Molecular Pharmacology and Experimental Therapeutics, Mayo Clinic, Rochester, MN, United States; ^5^ Division of Pediatric Cardiology, Department of Pediatric and Adolescent Medicine, Mayo Clinic, Rochester, MN, United States

**Keywords:** pediatric congenital heart disease, RNA sequencing, transcriptomes, animal model, iPSC models, gene expression, noncoding transcriptomes

## Abstract

Congenital heart disease (CHD) are genetically complex and comprise a wide range of structural defects that often predispose to - early heart failure, a common cause of neonatal morbidity and mortality. Transcriptome studies of CHD in human pediatric patients indicated a broad spectrum of diverse molecular signatures across various types of CHD. In order to advance research on congenital heart diseases (CHDs), we conducted a detailed review of transcriptome studies on this topic. Our analysis identified gaps in the literature, with a particular focus on the cardiac transcriptome signatures found in various biological specimens across different types of CHDs. In addition to translational studies involving human subjects, we also examined transcriptomic analyses of CHDs in a range of model systems, including iPSCs and animal models. We concluded that RNA-seq technology has revolutionized medical research and many of the discoveries from CHD transcriptome studies draw attention to biological pathways that concurrently open the door to a better understanding of cardiac development and related therapeutic avenue. While some crucial impediments to perfectly studying CHDs in this context remain obtaining pediatric cardiac tissue samples, phenotypic variation, and the lack of anatomical/spatial context with model systems. Combining model systems, RNA-seq technology, and integrating algorithms for analyzing transcriptomic data at both single-cell and high throughput spatial resolution is expected to continue uncovering unique biological pathways that are perturbed in CHDs, thus facilitating the development of novel therapy for congenital heart disease.

## 1 Introduction

With a prevalence of 6–13 in 1,000 newborn babies and approximately 261,247 infant deaths recorded globally, congenital heart diseases (CHDs) remain the most common and severe birth defect (Hoffman and Kaplan, 2002; Reller et al., 2008; Leirgul et al., 2014; GBD, 2017 Congenital Heart Disease Collaborators, 2020; Bakker et al., 2019; Liu et al., 2019). Based on their clinical manifestation, there are three types of CHDs: left-sided obstruction defects, septation defects, and cyanotic heart disease (Li et al., 2014). Left-sided obstructive lesions include hypoplastic left heart syndrome (HLHS), mitral stenosis, aortic stenosis, aortic coarctation, and IAA. Septation defects mainly affect the separation of the atria (atrial septal defects, ASDs), the ventricles, (ventricular septal defects, VSDs), or both (atrioventricular septal defects, AVSDs). Cyanotic heart diseases include Tetralogy of Fallot (TOF), transposition of the great arteries (TGA), tricuspid atresia, pulmonary atresia, Ebstein’s anomaly of the tricuspid valve, DORV, persistent truncus arteriosus (PTA) and anomalous pulmonary venous connection (Bruneau, 2008).

Diagnostic capabilities and advanced surgical intervention during infancy have dramatically improved over the past decades, improving survival for several patients with CHD who survive into adulthood (McCraken et al., 2018). However, those patients have an increased risk of heart failure and other non-cardiac mortality such as diabetes and cancer through poorly understood mechanisms (Hsu and Pearson, 2009; Ntiloudi et al., 2016; Barrett-Connor et al., 2018; Friedberg and Reddy, 2019).

Next-generation sequencing (NGS) technologies have revolutionized medical research and drastically improved our understanding that genetics has a crucial role in the pathogenesis of CHDs. Whole-genome and exome sequencing analyses facilitated the identification of myriads of genes encoding transcription factors and regulatory genes related to cardiac development (Fahed et al., 2013; Morton et al., 2022). Multiple lines of evidence have also provided a robust indication that a diverse array of genes and genetic aberrations are the main drivers of a majority of CHDs (Iascone et al., 2012; Theis et al., 2015a; Theis et al., 2015b; Zaidi and Brueckner, 2017). The complex anatomy and physiology of CHDs have hindered the development of animal models of CHDs which in turn has hampered progress in the development of novel therapies and explicitly understanding the biological mechanisms of CHDs. Even so, by combining powerful genetic tools with animal models, coupled with the need for more in-depth molecular studies, researchers have and still are uncovering multigenetic backgrounds and complex molecular networks underlying the pathogenesis of CHD. As an illustration, the first study to simulate HLHS, the most severe type of CHD, deployed a mouse-forward genetic screen approach to isolate mutant mice with a hypoplastic LV. In this study, mutations not previously identified were validated by CRISPR–Cas9 genome editing in both mice and zebrafish. Further, RNA-seq analysis of the hypoplastic LV from these mice found changes in gene expression patterns related to metabolism and mitochondrial pathways, calcium signaling, cardiac muscle contraction, and dilated/hypertrophic cardiomyopathy, similar to what has been shown in human SV myocardium-based analysis, advancing our understanding of the complex genetics and mechanisms associated with SV physiology (Liu et al., 2017).

With the popularity of NGS technologies, bulk RNA sequencing (bulk RNA-seq) has generated a huge amount of data about transcriptomic alterations in congenital heart diseases (Kaynak et al., 2003; Bittel et al., 2011; Grunert et al., 2016; Zhang et al., 2021). Recently, by deploying a multi-omics approach (single-cell RNA sequencing and single nucleic RNA sequencing), the pioneering study of Hill and others discovered distinct transcriptomes from 10 pediatric CHD donor tissues, providing more insight into understanding cardiac tissue molecular biology (Hill et al., 2022). Despite several efforts in understanding the cardiac transcriptomes even at the cellular level, only a subset of CHDs transcriptomes has so far been characterized, and many particularly, the non-coding transcriptomes exhibit high spatial and temporal expression patterns and are emerging as key drivers, regulators of differentiation, development, and disease pathology. Such important spatial and temporal expression patterns are better explored and delineated by deploying even more advanced transcriptome sequencing technology such as spatial transcriptomic sequencing (Zhu, 2016; Rao et al., 2021).

In this article, we review transcriptome studies of CHDs in humans and a variety of model systems, pinpointing promising transcriptomes that are potential diagnostic biomarkers and therapeutic targets while identifying gaps in the literature for future studies. We highlight the potential of advanced transcriptome sequencing technology in studying key functional biological mechanisms in CHDs and propose perspectives for future multi-omics work that could uncover novel treatment options for CHDs. Delineating the transcriptome in CHDs in finer resolution and defining them would improve our understanding of the morphological consequences of damaging variants and the cellular deficits that contribute to lifelong adverse events in patients with CHD, and also provides the opportunity to select new, specific, and more effective therapy to treat CHDs.

## 2 Role of genes in the development of CHD

**FIGURE 1:**
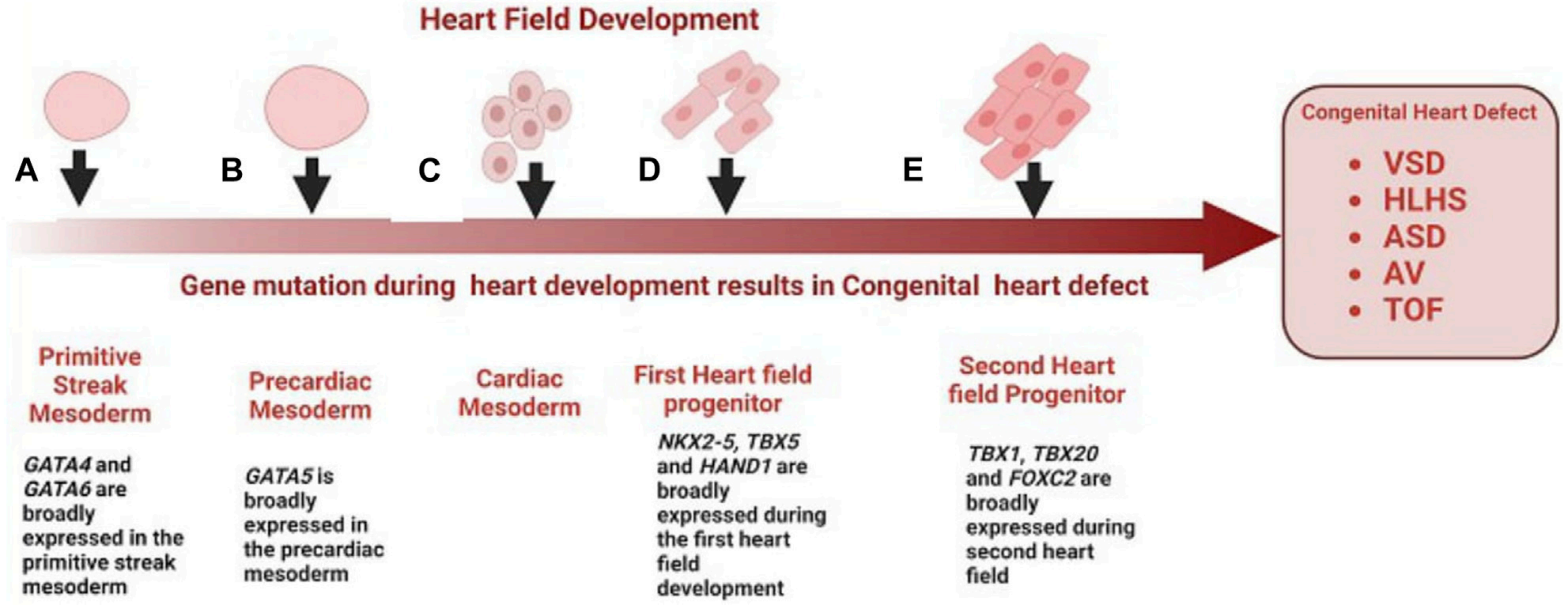
Role of genes in the development of congenital heart disease. **(A)** GATA4 and GATA6 are broadly expressed in the primitive streak mesoderm, mutations of GATA4 and GATA6 are a well-characterized cause of VSD **(B)** GATA5 is broadly expressed in the precardiac mesoderm, mutation in GATA5 have been associated with BAV and AV block **(C)** Cardiac mesoderm speciation give rise to first cardiac progenitors **(D)** First cardiac progenitors, NKX2-5, HAND1 and TBX5 are expressed at the earliest stages of cardiogenesis before the onset of myogenic differentiation, mutation in these genes during early cardiogenesis result is associated with ASV, VSD and TOF respectively **(E)** FOX transcription factors and TBX20 are expressed in the second heart field progenitor and throughout the developing heart, mutation of FOXF1, FOXC2 and TBX20 is associated with HLHS, and TOF.

Congenital heart diseases arise from abnormal heart development during embryogenesis. The earliest cardiac progenitors arise from lateral plate mesoderm and are controlled by a cascade of interacting transcription factors (Srivastava, 2006). Some notable genes expressed during heart field development include: *NKX2-5, TBX, HAND, NOTCH*, and *GATA* family, and several *FOX* transcription factors (Figure 1). *NKX2-5* is expressed at the earliest stages of cardiogenesis before the onset of myogenic differentiation, regulating cardiomyocyte differentiation and proliferation (Komuro and Izumo, 1993). *NKX2-5* mutations are known to be associated with AV block and ASD (Benson et al., 1998). *GATA4* and *GATA6*, are expressed broadly in the primitive streak mesoderm (Heikinheimo et al., 1994), while *GATA5* has a more restricted expression pattern in the precardiac mesoderm (Morrisey et al., 1997). Most heart defects associated with impaired conduction are observed in individuals with mutations in GATA6, whereas GATA4 mutations more often result in septal defects and endocardial cushion defects, but not issues with conduction (Ang et al., 2016). The TBX transcription factors are expressed throughout the developing heart, especially in the developing inflow tract, atrial wall, atrial septa, and atrioventricular (AV) endocardial cushions and they play a key role in regulating cardiomyocyte identity (Li et al., 1997). Mutations in TBX20 have also been associated with CHD such as TOF. Several forkhead box (FOX) transcription factors also play important roles in heart development, with mutations leading to vascular and cardiac defects and embryonic lethality in mice and are also commonly associated with the clinical processes of congenital heart in humans (Zhu, 2016). Mutations in FOXF1, FOXC2, and FOXL1 are well-characterized causes of TOF and HLHS (Stankiewicz et al., 2009). *HAND1* and *HAND2* are basic helix–loop–helix (bHLH) transcription factor genes that are excellent ventricular identity markers; their expression changes correlate with altered cardiac morphogenesis (George and Firulli, 2019). A mutation in HAND2 has been associated with VSD. Many other transcription factors have also been shown to cause CHD when mutated, and the phenotypes resulting from this mutation have been reviewed here (Williams et al., 2019).

## 3 Model systems for recapitulating human CHD

**FIGURE 2:**
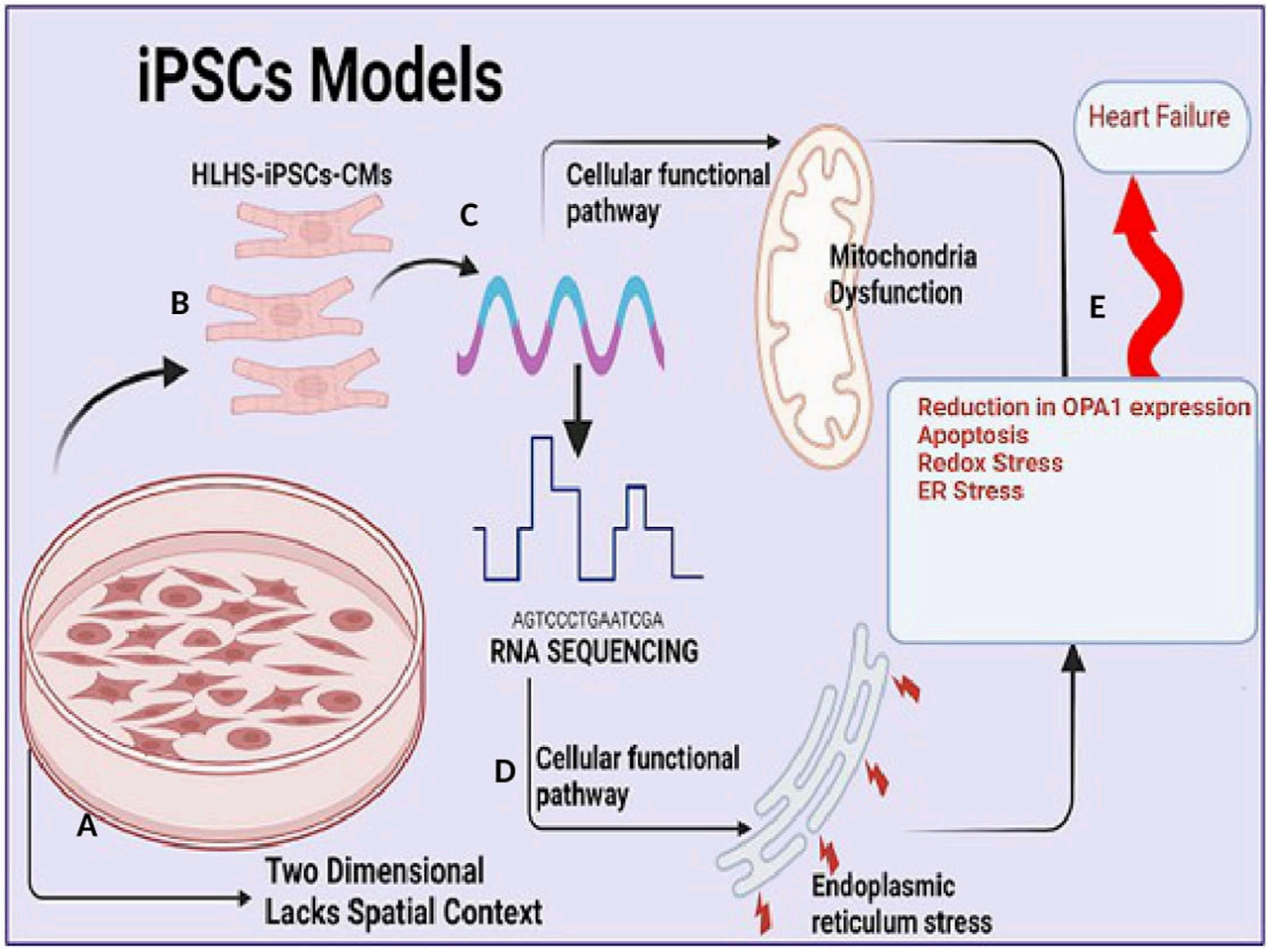
Induced pluripotent stem cell (iPSc) model in understanding congenital heart disease **(A)**. Induced pluripotent stem cell (iPSc) model allows for large scalability of cardiomyocytes and mimics human biological mechanism but they are two dimensional and lacks spatial context **(B)**. Cardiomyocytes (CM) cells from HLHS patients are generated and expanded from induced pluripotent stem cells (iPSc) via standardized protocol utilizing broad-spectrum pharmacological inhibitors **(C)**. Extraction of RNA, total/bulk RNA or single cell sequencing of cardiomyocytes. **(D)**. RNA sequencing analysis capable of identifying functional, biological pathway and signaling transduction involved in regulating mitochondria function and endoplasmic stress. **(E)**. Reduction in Optic atrophy 1 (OPA1) expression (a mitochondria fusion protein) results in increased apoptosis (an important mechanism of cell death in heart failure (HF), redox stress and endoplasmic reticulum stress which predisposes to adult heart failure.

Following the widespread acceptance of the genetic theory of diseases, the presumption that diseases and specific traits are caused by genetic variations has remained one of the most firmly upheld doctrines of medicine. The central dogma of molecular biology which suggested that genetic information flows primarily from nucleic acids in the form of DNA and RNA to functional proteins has brought light to the mechanisms governing the specification and transmission of genetic traits (Crick, 1970). Over the past decade, there has been an increasing interest in understanding the role of genetic variation in complex traits and human disease. However, the molecular mechanisms by which this genetic variation predisposes individuals to disease are still limited, impeding the development of therapeutic interventions. Modeled after the Human Genome Project, the NIH Genotype-Tissue Expression (The GTEx Consortium Science, 2015) Project was designed to explicitly delineate the molecular biology of tissues including genetic variation, gene expression, and other molecular phenotypes in multiple human tissues thus providing useful insights into understanding the role of transcriptomes across various mammals and pinpointing the functional interpretation and insights into disease etiology in a large scale (GTEx Consortium, 2013). The National Human Genome Research Institute (NHGRI) Mouse Transcriptome Project and Mammalian Gene Collection initiative focused on generating transcriptomes and a reference expression database for the C57BL/6 J mouse further describing the important role of animal model in understanding biological complexities of diseases (Strausberg et al., 1999; Li et al., 2017). Transcriptomic analysis of cardiovascular disease has been described in a variety of model systems ranging from iPSCs, rodents (Rao et al., 2022), pigs, non-human primates, chicken embryos, and zebrafish. In recent years, the development of a variety of model systems for CHD has opened up novel frontiers in understanding the molecular basis of CHDs. Human CHDs can never be perfectly emulated in pre-clinical animal models, however since early heart failure is a common long-term complication of several types of CHDs. Animal models of right heart failure, cardiac hypertrophy, and right ventricle pressure overload are often utilized to study CHD development (Diller et al., 2015; Liu et al., 2017). For instance, murine models of RV hypertrophy and chronic RV volume overload have improved our understanding of RV-specific adaptions and single ventricle-CHDs (Urashima et al., 2008; Reddy et al., 2012; Reddy et al., 2013; Blakeslee et al., 2017). In a pulmonary blood (PBF) shunt ovine model of CHD, Tian, and others traced “angiogenesis burst” between 1 and 4 weeks of age and described how disordered angiogenesis is implicated in pulmonary vascular remodeling secondary to congenital heart diseases (CHD) (Tian et al., 2011). Mouse models of CHD represent many aspects of human cardiac development as these models maintain the anatomical arrangement of the heart, and thus provide incredible insight into the role of specific genes and cell types that contribute to proper cardiogenesis. However, mutations modeled in mice often do not recapitulate many aspects of the human phenotype**.** *Drosophila* is another animal model of choice for human cardiac disease gene testing (Schroeder et al., 2019; Vissers et al., 2020; Souidi and Jagla, 2021) because of its low cost and high throughput. However, the classical fly mutants from forward genetic screens are typically loss-of-function alleles therefore fly mutations or some transcriptional factors may not be precise representations of human mutant transcripts (Koon and Chan, 2017). hPSC-based CHD models are highly scalable human cellular models of CHD used to study the mechanisms underlying cellular defects occurring in CHDs. However, while hPSC-based CHD models are highly scalable human cellular models of CHD, which are more likely to capture human-specific biology they lack anatomical structure, these models are two-dimensional and lack the spatial context of the human heart, which is critical for studying structural defects of CHDs (Figure 2). Though expensive large animal models such as pig and non-human Primates provide a more promising and more efficient mammalian *in vivo* model system to identify key genes and mechanisms critical for heart development and function that served as prototypes for mammalian studies due to the high degree of conservation of genetic pathways and reduced genetic complexity. In particular, non-human Primates because of their evolutionary proximity to humans, the similarity of the two species’ cardiovascular systems can be considered a better candidate to model heart disease. Many CHDs are caused by mutations in early cardiac TFs such as GATA4, TBX5, and NKX2-5, which steer broad gene expression programs leading to changes in cell identity. Understanding how these transcriptional changes from various models’ systems can alter the transcriptional and epigenomic landscape requires multi-omic approaches, such as single-cell RNA-seq, single nucleus, and total and spatial transcriptomes which are described in subsequent sessions.

## 4 Transcriptomes in congenital heart diseases

### 4.1 Transfer RNA fragments: a predictive biomarker for CHD

The rapid development of high-throughput sequencing technologies has revealed that most regulatory RNAs function without involvement in protein translation (Li and Liu, 2019). Beyond the central dogma of molecular biology lies the noncoding transcriptome, in which, unlike messenger RNA (mRNA), transcribed RNA is not translated into protein. Such noncoding RNAs (ncRNAs) comprise a sizable fraction of transcriptome by mass and play crucial roles in human biology and disease (Pallazo and Lee, 2015). Coding RNAs/mRNA encodes protein to act as various components including enzymes, cell structures, and signal transductors. Transfer RNA (tRNA) are 76–90 nucleotides, that serve as the physical link between transcriptome and proteome (the mRNA and the amino acid sequence of proteins respectively) decoding information present in mRNA sequences into specific proteins (Peng et al., 2022). In cardiovascular diseases, a wide range of RNA modifications including m^6^A (N6-adenosine methylation), m^5^C (5-methylcytidin), Nm (2′-O-ribose-methylation), (pseudouridine), m^7^G (N7-methylguanosine), and m^1^A (N1-adenosine methylation) have been found in tRNA, rRNA, mRNA which are involved in metabolic syndrome, heart failure, coronary heart disease, and hypertension (Wu et al., 2019). Transfer RNAs (tRNA) are involved in gene expression regulation, protein synthesis, inhibiting translation, and signal transduction ((Xie et al., 2020). tRNA was first thought to be stable, however, recent developments in unbiased high-throughput sequencing facilitated the discovery of a new class of tRNA: tRNA halves (31–40 nucleotides) and tRNA-derived fragments (tRFs, 14–30 nucleotides) (Cole et al., 2009; Lee et al., 2009; Liao et al., 2010). While our current understanding of the functional role of transfer RNA fragment (tRF) in CHD is still in its infancy, the pioneering study of Lu et al., 2023 has shown that distinct tRNA fragments might serve as a biomarker for accurate diagnosis of CHD during pregnancy. Here, 18 tRFs/tiRNAs were selected as predictive biomarkers of CHDs. By further analysis, tRF-58:74-Gly-GCC-1 and tiRNA-1:35-Leu-CAG-1-M2 were validated as promising biomarkers of CHD (Lu et al., 2023). The onset and progress of CHD, mainly in early pregnancy, is a complex and relatively long-term process involving many genetic and epigenetic alterations. The recent study of Li and others is the only study that has explored tFRNA in CHD, future studies with large sample sizes would add another dimension to this concept and open up a new perspective to understanding the relationship between the change of tRFs/tiRNAs expression level and the time of pregnancy, the causal relationship between tRFs/tiRNAs expression level and the occurrence of CHD. Thus, additional evidence of the role of transfer RNA fragments in the pathogenesis of CHD is warranted.

### 4.2 Small nucleolar RNAs in congenital heart disease

Small nucleolar RNAs (snoRNAs) represent a class of regulatory RNAs responsible for telomerase activity and posttranscriptional maturation of ribosomal RNAs (rRNAs) (Caveille et al., 1996; Ganot et al., 1997). RNAs that are highly similar in structure and function to snoRNAs are the so-called small cajal body associated RNAs (scaRNAs), which assemble in cajal bodies to modify spliceosomal small nuclear (sno)RNAs (Jady et al., 2003; Cao et al., 2018; Bergstrand et al., 2022). Failure of sno/scaRNAs has been implicated in pathologies such as congenital heart anomalies, neuromuscular disorders, and various malignancies. More precisely, scaRNA has been shown to have a role in splicing, and defects in splicing may contribute to severe congenital heart anomalies (Patil et al., 2015). Current information on the functional role of sno/sna RNA in CHDs is thus scarce and only a short list of reports has provided evidence of the involvement of sno/sna in CHDs. The first attempts by Obrien et al., show that in TOF samples, the targeted nucleotides of differentially expressed snoRNAs were concentrated in the 28S, 18S ribosomal RNAs, and 2 spliceosomal RNAs (U2 and U6) (Obrien et al., 2012). They further observed splicing variants in 51% of genes in the myocardium from children with TOF, which are critical for cardiac development (Obrien et al., 2012). Using RNA-seq, Patil et al., identified 12 scaRNAs downregulated in the right ventricle of infants with TOF. However, these 12 scaRNAs affected targeted only U2 and U6 snRNAs (Patil et al., 2015). Consistent with this, the snoRNAs that were downregulated in TOF samples in the work of Ciao and others targeted the spliceosomal RNAs of the U2 spliceosome (Cao et al., 2018). Small Nucleolar RNA have been identified in CHDs caused by septation defects with a high incidence reported in VSD. Small Nucleolar RNA Host Gene 6 (SNHG6) is highly expressed in fetal cardiac tissues of VSD patients (Jiang et al., 2019). While experimentally testing differentially expressed genes they demonstrate that SNHG6 gain-of-function simultaneously blocked cardiomyocyte proliferation while enhancing apoptosis. Although the mechanism by which SNHG6 blocks cardiomyocyte proliferation is not known with certitude, SNHG6 contributes to ventricular septal defect formation via negative regulation of miR-101 and activation of Wnt/β-catenin pathway, suggesting a plausible mechanistic link between SNHG6 upregulation, impaired miR-100 expression, Wnt/β-catenin activation and the formation of VSD. Overall, these observations suggested a link between levels of snoRNA that target spliceosomal RNAs, spliceosomal function, and heart development and translation of developmentally important gene that possibly contributes to the cardiac defect.

### 4.3 Long noncoding RNA in congenital heart disease

**FIGURE 3:**
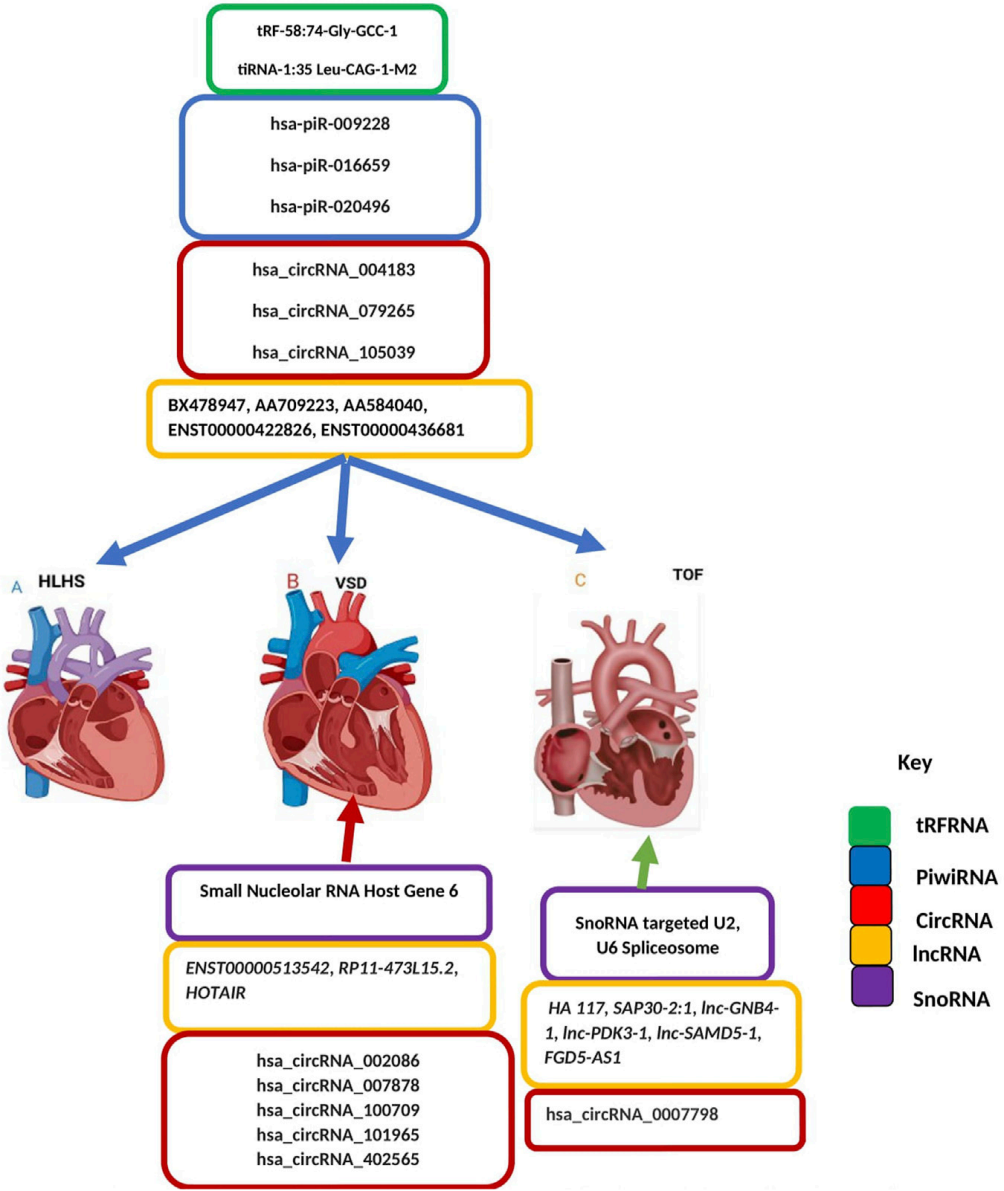
Non-coding Transcriptomes in Congenital Heart Disease. Colored boxes indicate class of non-coding RNA across each type of CHD as represented on the key **(A)** Left-sided obstructive lesions CHD **(B)** Septation defects CHD **(C)** Cyanotic heart diseases CHD. Blue arrow describes transfer RNA, Piwi RNA, Circular RNA, long noncoding RNA and small nucleolar RNA associated with all types of CHD including left-sided obstructive lesions CHD, septation defects CHD and cyanotic heart diseases CHD. Red an-ow indicates circular RNA, long noncoding RNA and small nucleolar RNA associated with septation defect CHD. Green arrow indicates circular RNA, long noncoding RNA and small nucleolar RNA associated with Cyanotic heart diseases CHD.

Long-noncoding RNA is a heterogeneous group of non-coding transcripts more than 200 nt long that are involved in many biological processes. This class of ncRNA makes up the largest portion of the mammalian non-coding transcriptome (Mercer et al., 2009). Recent studies suggest that circulating plasma lncRNAs have significant potential as a novel diagnostic biomarker in predicting CHDs. Gu et al. (2016) identified five differentially expressed lncRNAs- ENST00000436681, ENST00000422826, AA584040, AA709223, and BX478947 across fetal CHD samples and controls suggesting that circulating plasma lncRNA may serve as novel biomarkers for CHD diagnosis (Gu et al., 2016). As aptly demonstrated (Figure 3), another line of evidence supporting the role of lncRNAs as promising biomarkers for CHDs is the detection of the high expression level of HOTAIR in right atrial biopsies of patients with ASD and VSD (Jiang et al., 2019), the validation of two candidate lncRNAs, ENST00000513542 and RP11-473L15.2 significantly associated to VSD and ASD (Song et al., 2013), and more recently the identification of a MALAT1 polymorphism associated to VSD and ASD (Li et al., 2018). Besides the role of lncRNAs in cardiac septal defect, it is hypothesized that high HA117 expression is associated with adverse outcomes in TOF patients (Wang et al., 2018). However, the mechanism of action of HA117 in TOF remains unclear. Additional studies are required to fully elucidate the functional relevance of lncRNAs in this context. Interestingly a novel lncRNA, SAP30-2:1 with an unknown function was significantly downregulated in damaged cardiac tissues from patients with CHD. Further, the knockdown of SAP30-2:1 decreased the expression of the HAND2 gene suggesting that SAP30-2:1 may be involved in heart development by targeting HAND2 and may thus represent a novel therapeutic target for CHD (Ma et al., 2021). More work is required to understand the function mechanism of lncRNA SAP30-2:1 in TOF.

### 4.4 Micro RNA in congenital heart disease

**FIGURE 4:**
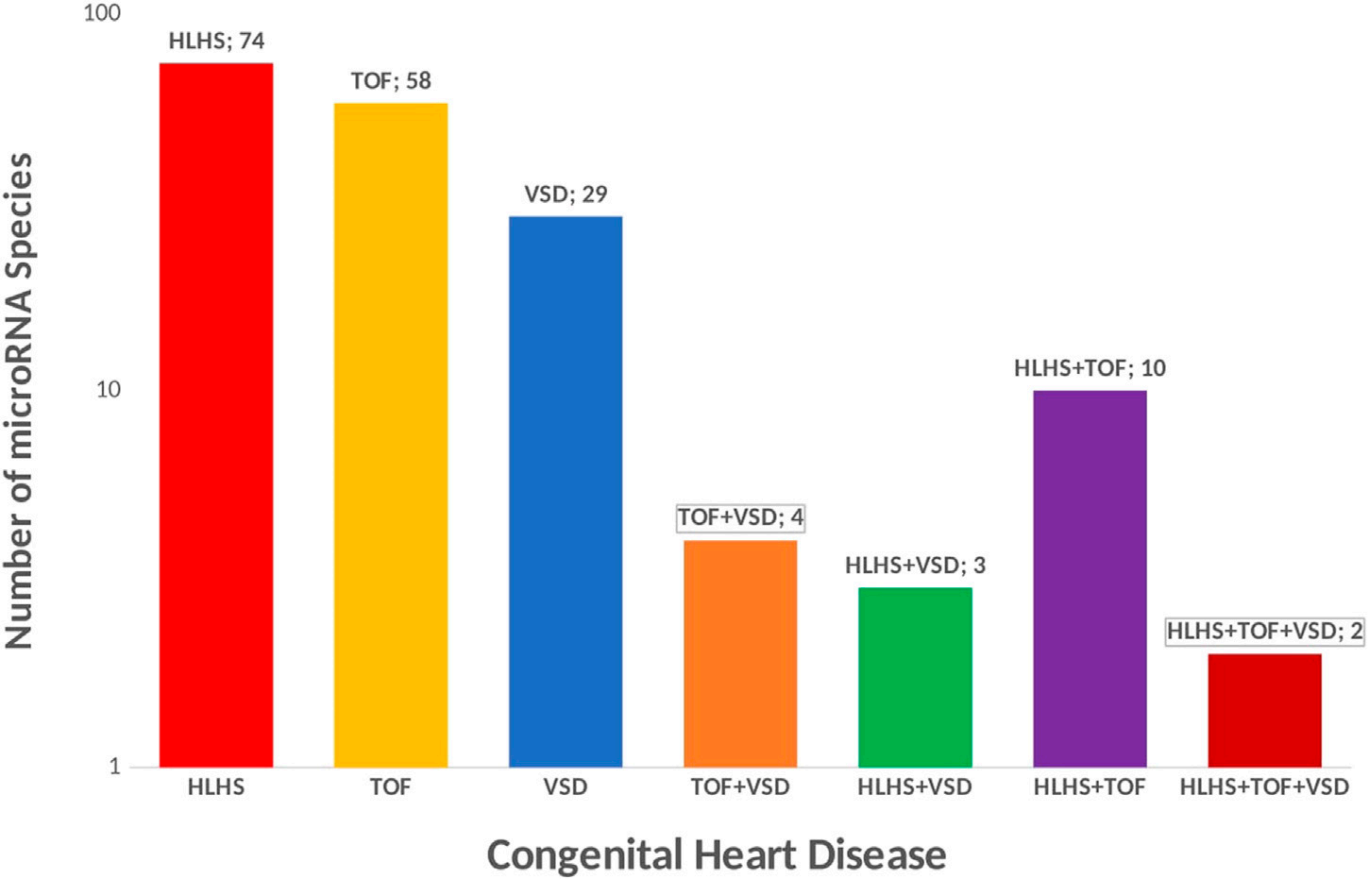
Micro RNA in Congenital Heart Disease. Micro-RNA species in CHDs could be potential biomarker for treatment. 74 unique microRNA species have been reported in HLHS, 58 unique microRNA species have been reported in TOF, 29 unique microRNA species have been reported in VSD, 4 micro-RNA species are commonly shared across CHD associated with septation defect and cyanotic heart disease (TOF and VSD), 3 micro-RNA species are commonly associated with HLHS and VSD, 2 micro-RNA species are commonly associated with HLHS, TOF and VSD.

***miRNAs*** are small ncRNAs of 21- to 23-nucleotide that mediate post-transcriptional gene silencing by controlling the translation of mRNA into proteins through binding to complementary sites predominantly in the 3’ untranslated regions (UTR) of pre-messenger RNA (mRNA, protein-coding) (He and Hannon, 2004; Mendell, 2005; Treiber et al., 2019). A concise summary of micro-RNA in congenital heart disease has been reviewed elsewhere (Duenas et al., 2019). The majority of microRNA have been implicated in left-sided obstruction defects especially hypoplastic left heart syndrome (Figure 4)**,** yet a reference micro-RNA diagnostic biomarker has not been confidently used in the clinical setting due to a limited understanding of the functional mechanism. In HLHS, three microRNA species (miR-99a, miR-100, and miR-145a) were downregulated immediately after the stage 1 operation of the Norwood procedure while values returned to control levels after stage 3 operation, indicating a strong influence of altered blood flow conditions on microRNA expression. Further, the downregulation of micro RNAs in the RV of HLHS patients, directly regulate the expression of FOG2 that modulates the expression of *GATA4*, *GATA5*, and *GATA6*, key players in cardiac development (Sucharov et al., 2015) The most relevant microRNA in CHDs is miR-1 (van Rooij and Olson, 2007; Wei et al., 2014). miR-1 simultaneously targets the cardiac transcription factor HAND2 to **negatively control cardiogenesis** and also targets histone deacetylase (HDAC) 4 to inhibit downstream MEF2 and several other regulators of cardiac growth during development leading to complex heart defects (van Rooij and Olson, 2007; Bruneau, 2008; Wei et al., 2014). In addition to experimental evidence, microRNAs are attractive clinical biomarkers as they remain stable in blood, urine, and other biological fluids and evade RNA-degrading enzymes. By identifying four significantly upregulated microRNAs (miR-19, miR-22, miR-29c, and miR-375) in mothers carrying fetuses with CHD. Yu et al. (Yu et al., 2011; Zhu et al., 2013., demonstrated, that microRNAs in maternal serum are candidate biomarkers for prenatal detection of fetal CHD in early pregnancy. Both studies also demonstrated that miR-19b and miR-29c were significantly upregulated in VSDs and all four microRNAs upregulated in TOF. Several studies have shown there is a genetic association between microRNA and the occurrence of TOF (Bittel et al., 2011; Obrien et al., 2012; Zhang et al., 2013; Liang et al., 2014; Huang et al., 2015; Low et al., 2015; Abu Halima et al., 2017; Lai et al., 2017; Grunert et al., 2019), however, the functional mechanism by which these micro-RNAs are upregulated in TOF remains to be elucidated.

### 4.5 Circular RNA in congenital heart disease

Similar to miRNAs and long noncoding RNAs (lncRNAs), circRNAs are becoming a new research hotspot in the field of RNA and could be used as a predictive tool to understand the biological mechanism of several CHDs. CircRNAs are single-stranded and created by back splicing of 3′-5′ linear coding or non-coding RNAs, forming covalently closed loops (Carrara et al., 2018). Seminal studies in VSD and TOF patients identified CircRNAs as biomarkers supporting their use as predictive tools. Liu and others identified 20 differentially expressed circRNAs in VSD and recommended 5 circular RNAs differentiated expressed in VSD myocardial tissues compared with controls. hsa_circRNA_002086 was highly expressed and hsa_circRNA_007878, hsa_circRNA_100,709, hsa_circRNA_101,965, hsa_circRNA_402,565 *were underexpressed in VSD sample*s (Liu et al., 2018). Yu et al. provided a comprehensive understanding of the circular RNA network involved in the biology of TOF. In their work, 276 differentially expressed circRNAs; 214 upregulated and 62 downregulated circRNAs were identified in TOF samples. Additional assay validated hsa_circRNA_0007798 as a significant biomarker and therapeutic target for TOF (Yu et al., 2021). Seven differentially expressed circRNAs were identified in CHD patients. Among these 7 circRNA, hsa_circRNA_004183, hsa_circRNA_079265, and hsa_circRNA_105,039 were under-expressed in plasma from children with CHD (Wu et al., 2019). These circRNAs may be crucial in the development of CHD and may serve as novel non-invasive biomarkers for the diagnosis of CHD in children. Interestingly, individual circRNAs have a unique characteristic of inhibiting microRNAs (miRs), by binding/sponging microRNAs, thereby regulating the corresponding miR target genes (Hansen et al., 2013; Morten et al., 2022). In a recent study, hsa_circ_105,039 acted as a sponge for miR-17. miRNAs possess positive effects on the progression of CHDs, therefore by acting as a sponge for miR-17, hsa_circ_105,039 may be a promising biomarker for prognosis and therapeutic target for CHD (Yu t al., 2021). Most recently, hsa_circ_0003416 was found to be significantly downregulated in children with pulmonary arterial hypertension associated with congenital heart disease (PAH-CHD), through a poorly understood mechanism (Huang et al., 2022). Collectively, these studies provide some fundamental data that suggest the important role of circRNAs in heart development and their potential as promising predictive tools and therapeutic targets for CHD. Additional studies are needed to elucidate the functional and biological mechanism of circRNAs in the development of CHDs.

### 4.6 PIWI-interacting RNAs in congenital heart disease

PIWI-interacting RNAs (piRNAs) are small RNAs, 24–31 nucleotides in length with big functions, they associate with PIWI proteins to form the Piwi-piRNA pathway which mediates epigenetic programming and posttranscriptional regulation (Iwasaki et al., 2015). To date, only a limited number of reports have provided evidence of the differential expression of piRNAs in CHDs (Jia et al., 2021). Here, they identified a biomarker panel of three pregnancy-associated exosomal piRNAs (hsa-piR-009228, hsa-piR-016659, and hsa-piR-020496) which distinguished fetuses with congenital malformations from normal fetuses. However, whether they can serve as *bona fide* biomarkers for all classes of CHDs remains to be elucidated. Other types of ncRNAs whose biological functions are poorly defined include promoter-associated small RNAs (PASRs) (Kapranov et al., 2007), TSS-associated RNAs (TSSa-RNAs) (Seila et al., 2008), promoter upstream transcripts (PROMPTs) (Preker et al., 2008) and transcription initiation RNAs (tiRNAs) (Taft et al., 2009). No study has provided evidence of a genetic association between them and the occurrence of CHD.

## 5 Transcriptome sequencing technology

### 5.1 Insights from total RNA sequencing

The discovery of genes and variants with a potential causal link to CHD combined with analyses of gene expression during development and functional studies in model systems provide crucial evidence for assigning causality in the clinical setting (Richards et al., 2015; Strande et al., 2017). With the popularity of sequencing technologies, RNA sequencing of tissue samples (bulk RNA-seq) has generated a huge amount of data about transcriptomic alterations in cardiovascular diseases (Wang et al., 2009; Ramachandran et al., 2022). Pediatric tissue samples are rare and exceedingly difficult to obtain (Brisson et al., 2012) this has hampered or slowed down the tissue molecular biology of CHDs, limiting a thorough understanding of the biological mechanism of CHDs. Samples assayed recently were explanted cardiac tissue from heart transplant patients and donor samples (Hill et al., 2022). Two major complicating factors when analyzing human heart tissue (compared to other human tissue types) are that it contains a large proportion of fibrous tissue and has a low cell density, so disrupting the cells and extracting their total RNA is challenging. Thus, rigorous precautions must be taken to avoid degradation of RNA during its extraction, and (hence) impairment of both RNA quality and yields. Detailed RNA-seq studies of healthy and failing human myocardium have revealed remarkable similarities between upregulated genes in the failing heart and fetal myocardium (Akat et al., 2014). Therefore, considering the complication of procuring pediatric tissue to study congenital heart disease, the inability to create a perfect CHD model, and the fact that heart failure is a major long-term complication of CHD. Researchers could gain more insight into the biological mechanism of CHD by creating heart failure surgical models. Pathway analysis approach has been employed to study RNA seq data, investigate enrichment for biologically relevant pathways and functions and pinpoint differentially expressed genes in CHD. Our cross-study comparison based on data from several CHD cohorts describes some definitive transcripts, prominent differentially expressed genes, and enrichment pathways in CHD by applying bulk RNA sequencing technology (Table 1).

**TABLE 1:** Notable Genes and Functional Pathway associated with congenital heart disease in Human Patients.

Model	Biological sample	Total DEGS	Signaling pathway	Notable Genes/DEGs	References
**Human**	Blood samples from 35 CHD (ASD and VSD) patients	FOXP1 and ADAR2 were downregulated while ADAR1 was upregulated in CHD patients	Cardiac homeostasis, cardiac fibrosis, hypertrophy, and cardiomyocyte proliferation	ADAR 2, FOXP1, ADAR 1	Altaf et al. (2019)
**Human**	Right Ventricle heart tissues from 22 TOF patients	41 genes with differential expressions were reported as CHD-related genes	Blood vessel morphogenesis and other CHD-related signaling pathways such as extracellular matrix assembly, Wnt, BMP, and ERK, and disease ontologies (e.g., cardiovascular disorders and non-cardiac disease)	WNT3, SOX9, PEX19, VIT, CDH11, IGFBP5, HAS2, ENO2, EGR1, NRAS, PTEN, and SMAD4	Zhang et al. (2021)
**Human**	Cardiovascular tissue (right ventricle, pulmonary valve, and pulmonary artery) from 19 children with TOF	715 upregulated genes and 347 downregulated genes	WNT and NOTCH pathways have several members with significantly altered expressions	DVL3, WNT5B, DTX3	Bittel et al. (2011)
			Biofunction networks identified were protein synthesis, cardiovascular disease, genetic disorder, neurological disease, and cell death	NPPA	
**Human**	RV tissue of TOF patients	33 upregulated and 8 downregulated genes	Pathways Leading to cardiac dysdevelopment	KCNJ2, FBN2, SLC38A3 and TNNI1	Grunert et al. (2019)
			BMP signaling		
**Human**	RV tissue of TOF patients	SNIP, A2BP1, and KIAA1437 were upregulated, and genes markedly downregulated included STK33, BRDG1, and TEKT	Cell motility	SNIP, A2BP1 KIAA1437, STK33, BRDG1, and TEKT	Kaynak et al. (2003)
			Developmental processes		
			Calcium binding		

### 5.2 Insights from single-cell transcriptomics

The advent of single-nuclear RNA sequencing and single-cell RNA sequencing (scRNA-seq) technology has enabled a detailed characterization of the many cells that populate the human mature heart (Cao et al., 2019; Litvinukova et al., 2020). Both technologies are outstanding approaches to exploring transcriptome dynamics at the resolution of a single cell (Shapiro et al., 2013) and identifying transcriptional heterogeneity between different cell lineages and distinct cell states. The healthy hearts in human and mouse reveals that heart tissue is typically composed of a variety of cell types, including but not limited to four subsets of ventricular cardiomyocytes and five subsets of atrial cardiomyocytes endothelial cell, fibroblast, macrophages, and smooth muscle cell (Litvinukova et al., 2020; Tabula Muris Consortium, 2020; Wang et al., 2021). Genes of the Notch signaling pathway are highly enriched in endocardial cells at gestational week 7, when compaction of the myocardium occurs, whereas genes related to the BMP pathway are expressed in endocardial and fibroblast-like cells from gestational week 5 to week 25, reflecting periods of endocardial-to-mesenchymal transition (Cui et al., 2019). Single-cell RNA sequencing provides a powerful tool to study DEG profiles in the cell subpopulations of interest at the single-cell level. This could enhance the understanding of the underlying mechanisms of CHD at both the cellular and molecular levels and highlight potential targets for the treatment of CHD. However, only a few studies have yet investigated the development of various categories of CHDs using scRNA-seq analysis. Among them, scRNA-seq studies of heart development and gene expression signatures in cells derived from CHD and normal control tissues have mostly been based on animal models, and less so on human cardiac tissue. scRNA-seq studies of human fetal hearts, although more limited in scope than studies on adult hearts, have identified transcriptional signatures of cardiac muscle cells (*TNNI3* and *TNNT2*), fibroblast-like cells (*COL1A1*, *COL1A2*, and *POSTN*), endothelial cells (*PECAM1* and *KDR*) and valvular cells (*SOX9*) (Cui et al., 2019). With scRNA-seq analyses of healthy and diseased hearts, Gladka and others found that CKAP4 could modulate the activation of fibroblasts, showing positive correlations with known myofibroblast markers (Gladka et al., 2018). Yang et al. suggested that selective expression of NEXN, an F-actin-binding protein, could lead to ASD by inhibiting *GATA4* (Yang et al., 2014). Duong et al. showed that Nr2f1a is expressed in differentiated atrial cardiomyocytes and that it mediates the size of the atrial and atrial-atrioventricular canal by regulating the differentiation of atrial cardiomyocytes (Duong et al., 2018). Single-nucleus RNA sequencing analysis on 157,273 nuclei from control heart tissues and heart tissues from patients with hypoplastic left heart syndrome (HLHS), tetralogy of Fallot, and dilated and hypertrophic cardiomyopathies a recent study found CHD-specific cell states in cardiomyocytes, which showed evidence of insulin resistance and increased expression of genes associated with *FOXO* signaling and *CRIM1*. Cardiac fibroblasts in HLHS were enriched in a low-Hippo and high-YAP cell state characteristic of activated cardiac fibroblasts (Hill et al., 2022). scRNA-seq analysis offers a promising paradigm for the identification of functionally relevant pathways, validated markers, and therapeutic targets. A spectrum of changes associated with various cell subpopulations in ASD was captured in various clusters in the development of ASD. (Wang et al., 2021). Cells in cardiomyocyte clusters showed significantly higher expression of *FABP4, CD36, TNNT3*, and *AQP1,* which are markers of cardiomyocytes. Markers of endothelial cells, including *SELE, ACKR1, PLVAP, DNASE1L3*, and *CCL14,* were highly expressed in Cluster 5 also known as the endothelial cell cluster. Clusters 4 and 6 were considered smooth muscle cell clusters due to the high expression of markers *RGS5, GJA4, TAGLN, ACTA2, MYL9*, and *SOD3*. Markers of fibroblasts, including *DCN, COL1A2, LUM, COL1A1, FBLN1*, and *TCF21*, were highly expressed in Clusters 2, 3, and 7. Hence, Clusters 2, 3, and 7 were defined as fibroblast clusters. Cluster 8 was considered a macrophage cluster due to the high expression of markers *AIF1*, *CD163*, and *CD68*. ASD showed a decreased proportion of cardiomyocytes and an increased proportion of fibroblasts. There was more cellular crosstalk among cardiomyocytes, fibroblasts, and macrophages, especially between fibroblasts and macrophages. For all cell types, the majority of the DEGs were downregulated in ASD samples. For cardiomyocytes, there were 199 DEGs (42 upregulated and 157 downregulated) between ASD and normal samples (Wang et al., 2021).

### 5.3 Insights from spatial transcriptomics sequencing

Despite the many advantages of total/bulk RNA sequencing and scRNA-seq, there are several pitfalls. In standard bulk RNA-seq, whole tissue biopsies are homogenized, and only average representations of expression profiles within the entire sample are obtained. With single-cell transcriptomic sequencing, the coverage of gene expression quantification in scRNA-seq data (usually covering approximately up to 10,000 genes) is also substantially compromised in comparison with bulk RNA-seq data (usually covering 100,000 genes) (Hou et al., 2020) Further, scRNA-seq only isolate individual cells in droplets and does not preserve the tissue structure that is a fundamental component of every biological organism. As a result, information on spatial patterns of gene expression is lost and signals from subpopulations of cells with deviant profiles, such as those with low-level marker gene expression, are obscured.

To overcome these deficiencies, spatial transcriptomics (ST) technology (Ståhl et al., 2016) offers a promising paradigm enabling spatial analysis of important marker genes expressed at low levels within whole tissue sections. Spatial transcriptomic profiling provides the genomic information of single cells as they are intricately spatially organized within their native tissue environment (Li and Peng, 2022). Expanding our knowledge on the expression of transcripts meanwhile preserving the morphology of tissues will facilitate the understanding of cell type heterogeneity, cell-cell interaction, and cell fate dynamics in normal and abnormal biological contexts. As an illustration, hPSC-based CHD models which are more likely to capture Human-specific biology are two-dimensional and thus lack the spatial context of the Human heart, which is critical for studying structural defects of CHDs. Spatial transcriptomics technologies hold the promise of overcoming this limitation as transcriptomics expressions are captured while preserving spatial anatomy (Mantri et al., 2021). To the best of our knowledge, spatial mapping of transcriptional differences across ventricle and atrial tissue pediatric heart tissue in pediatric congenital heart disease has not been previously described. However, by combining single-cell and spatial transcriptomics, Stahl and others studied the development of the chicken heart from the early to late four-chambered heart stage and identified anatomically restricted expression patterns, including the expression of genes implicated in congenital heart disease. They also discovered a persistent enrichment of the small, secreted peptide, thymosin beta-4, throughout coronary vascular development and uncovered an intricate interplay between cellular differentiation and morphogenesis (Mantri et al., 2021). While SPTseq is gaining popularity in translational cardiovascular research and facilitating transcriptomics assays in the heart while preserving spatial anatomy, current spatial transcriptomics approaches lack single-cell resolution. A better approach to unraveling cellular interaction in spatially resolved gene expression entails combining both single-cell and spatial transcriptomic sequencing with algorithms for data integration. As explicitly described here (Mantri et al., 2021; Shi et al., 2022; Longo et al., 2021), spatial transcriptomics data can be integrated with the scRNA-seq data using Seurat-v3 anchor-based integration (Hie et al., 2019; Butler et al., 2018). These techniques along with the use of humans, animals, and the hPSC-based CHD model can help in understanding the development of CHD and pinpointing specific therapeutic targets.

## 6 Conclusion and future directions

Despite the progress of next-generation sequencing, there are still gaps in the literature regarding transcriptomic profiling in congenital heart disease and identifying consistent therapeutic markers. Relying on diagnostic biomarkers is a promising approach for the early detection of CHDs in infant blood. For example, transcriptome changes in the blood (cells or plasma) may help to better diagnose or determine the prognosis of patients. NcRNA signatures provide valuable molecular insight into patient phenotypes and could add to traditional markers and established clinical variables. LncRNA biomarkers are also being investigated as novel predictive tools to monitor therapeutic effectiveness and stratify patients. However, it cannot be excluded that there are still some CHDs that cannot be detected throughout pregnancy due to a lack of serum biomarkers for more comprehensive screening and diagnosis of CHD (McCracken et al., 2018). Understanding the functional mechanism of novel non-coding transcriptomes (**described in Figure 3**) could still lend a clue or open up avenues for deciphering diagnostic makers, especially for CHDs like HLHS that present with poor prognoses even after multiple surgeries. Numerous short-circulating miRNAs have been implicated in CHDs. miR-329, and miR-222 species commonly shared across all types of CHD are promising biomarkers to inform tailored treatment selection and monitor ongoing efficacy and thus deserve further investigation. Spatial transcriptomics technologies hold the promise of overcoming the limitations of single-cell transcriptomics as transcriptomics expressions are captured while preserving spatial anatomy (Mantri et al., 2021), as such its represent a powerful tool for researcher seeking to deploy embryo model for studying congenital heart disease. Additionally, generating 3D models in tissue organoids and generating a post-natal model of CHD are important topics for future research.

## Abbreviations

CHD, congenital heart disease; ER, Endoplasmic reticulum; OPA, optic atrophy type 1 (OPA1; bHLH, Basic helix loop helix; IAA, interrupted aortic arch (IAA); TOF, Tetralogy of fallot; HLHS, Hypoplastic left heart syndrome; VSD, Ventricular septal defect; PTA, persistent truncus arteriosus; DORV, Double outlet right ventricle; CRISPR, clustered regularly interspaced short palindromic repeat; NIH, National Institute of Health; PBF, pulmonary blood flow; hPSC, Human pluripotent stem **cells**; FFPE, Formalin fixed paraffin embedded; DEG, Differentially expressed genes; RIN, RNA integrity number; cDNA, complementary DNA; mRNA, messenger RNA; ST, Spatial transcripome; WNT, Wingless/Integrated’ signaling; LncRNA, Long coding RNA; Sno/Sna RNA, Small nucleolar RNA; ScaRNA, Small Cajal body-specific RNA; CircRNA, Circular RNA; MiRNA, Micro RNA; piRNA, Piwi interacting RNA; RV, Right Ventricle; LV, Left ventricle.
